# The Effects of Acute Exercise on Short- and Long-Term Memory: Considerations for the Timing of Exercise and Phases of Memory

**DOI:** 10.5964/ejop.2955

**Published:** 2021-02-26

**Authors:** Paul D. Loprinzi, Sierra Day, Rebecca Hendry, Sara Hoffman, Alexis Love, Sarah Marable, Elizabeth McKee, Sydney Stec, Hanna Watson, Brittney Gilliland

**Affiliations:** a Exercise & Memory Laboratory, Department of Health, Exercise Science and Recreation Management, The University of Mississippi, Oxford, MS, USA; University of South Wales, Pontypridd, United Kingdom

**Keywords:** cognition, cognitive function, exercise, long-term potentiation, physiscal activity

## Abstract

The specific questions addressed from this research include: (1) Does high-intensity acute exercise improve memory?, (2) If so, do the mechanisms occur via encoding, consolidation, or retrieval? and (3) If acute exercise occurs in multiple phases of memory (e.g., before encoding and during consolidation), does this have an additive effect on memory? Three experimental, within-subject, counterbalanced studies were conducted among young adults. High-intensity exercise involved a 20-minutes bout of exercise at 75% of heart rate reserve. Memory was evaluated from a word-list task, including multiple evaluations out to 24-hours post-encoding. The timing of the exercise and memory assessments were carefully positioned to evaluate whether any improvements in memory were driven by mechanisms related to encoding, consolidation, and/or retrieval. We demonstrated that high-intensity acute exercise enhanced memory. This effect was robust (repeatable) and occurred through encoding, consolidation and retrieval-based mechanisms. Further, incorporating acute exercise into multiple phases of memory additively enhanced memory function.

Previous experimental work has demonstrated that the acute exercise, including moderate- and vigorous-intensity exercise, may enhance episodic memory function (Brisswalter, Collardeau, & Rene, 2002; Chang, Labban, Gapin, & Etnier, 2012; Loprinzi, et al., 2019; Roig, Nordbrandt, Geertsen, & Nielsen, 2013; Tomporowski, 2003; Winter et al., 2007). However, as recently demonstrated via a systemic review (Loprinzi, 2018), higher-intensity acute exercise may be more beneficial in enhancing episodic memory. Further, as demonstrated elsewhere (Loprinzi et al., 2019; Roig et al., 2016), the timing of exercise plays a critical role in this acute exercise-memory relationship. That is, acute exercise occurring before memory encoding (Labban & Etnier, 2011, 2018) and during memory consolidation (van Dongen, Kersten, Wagner, Morris, & Fernandez, 2016) may enhance episodic memory,^1^1Episodic memory herein is referred to as a retrospective memory involving spatial-temporal aspects of the memory. whereas acute exercise during memory encoding may impair episodic memory function (Loprinzi, Day, & Deming, 2019).^2^2The three phases of memory referred herein include encoding, consolidation and retrieval. Encoding refers to the acquisition of the memory trace; consolidation refers to the stabilization of the memory trace; and retrieval refers to the reactivation of the memory trace to facilitate memory recall. Despite this evidence suggesting a temporal effect of acute exercise on episodic memory function, at this point, we need additional work evaluating whether the primary memory enhancement effects of acute exercise occurs through enhancing encoding, consolidation, or memory retrieval.

For example, from previous work on this topic, it is not certain as to whether the exercise bout occurring prior to memory encoding was effective in improving memory via encoding-based mechanisms, as this bout of exercise could have also induced residual (e.g., sustained physiological arousal) effects on consolidation. Similarly, previous work that has included the acute bout of exercise during the consolidation period often included this exercise bout close to when memory retrieval occurred; thus, this makes it difficult to determine whether the beneficial effects of acute exercise occurred through either consolidation or retrieval-based mechanisms (these underlying mechanisms have been discussed elsewhere; El-Sayes, Harasym, Turco, Locke, & Nelson, 2019; Loprinzi, Edwards, & Frith, 2017).

As such, the present set of experiments aims to help disentangle these issues. That is, we designed several conditions that integrated the acute bout of exercise either before encoding (with an extended delay period to minimize any potential effects on retrieval), during the early phase of consolidation (with an extended delay period to minimize any potential effects on retrieval), or during the later consolidation period shortly before memory retrieval (to potentially enhance retrieval-based mechanisms, such as inducing the allocation of mental resources to facilitate cue-based retrieval; Loprinzi et al., 2017).

Further, we also aimed to evaluate whether there may be an additive effect in enhancing memory if exercise occurs during multiple phases of memory. This has been speculated elsewhere (Roig et al., 2016) and tested in prior work (Loprinzi, Chism, & Marable, 2019; Slutsky-Ganesh, Etnier, & Labban, 2020). Such an additive effect is plausible given that, for example, acute exercise prior to encoding may enhance encoding-based mechanisms (e.g., enhanced psychological attention; discussed in detail elsewhere; Loprinzi, Ponce, & Frith, 2018), and acute exercise during consolidation may enhance consolidation-based mechanisms (e.g., CREB phosphorylation and long-term potentiation; discussed in detail elsewhere: El-Sayes, Harasym, Turco, Locke, & Nelson, 2019; Loprinzi et al., 2017, 2018), and together, they may act synergistically to enhance memory performance.

Specifically, Experiment 1 addresses these gaps in the literature by employing a 6-condition, within-subject design. A potential concern with Experiment 1 is that the consolidation period (i.e., 35-minutes)^3^3We initially selected a 35-minute consolidation period as past research has shown that exercise can improve long-term memory that involves a 20–30-minute consolidation period (Frith et al., 2017). may have been too short to conclude whether any effects of acute exercise on enhancing memory occurs via consolidation-based processes. As such, Experiment 2 extends this consolidation period out to 4-hours. Additionally, the optimal timing of exercise during the consolidation period is unknown, as previous work demonstrates that an acute bout of exercise occurring during the start of the consolidation period may enhance memory (Loprinzi et al., 2019), and similarly, other work also demonstrates that exercising during the later stages (i.e., 4 hours into consolidation) of consolidation may also enhance episodic memory (Delancey, Frith, Sng, & Loprinzi, 2018; van Dongen, Kersten, Wagner, Morris, & Fernandez, 2016). Experiment 3 evaluates whether an acute bout exercise during the middle periods of consolidation (e.g., 2-hours into consolidation) may enhance episodic memory function. Across these three experiments, our working hypotheses were that acute exercise would enhance memory, even when occurring in distinct memory phases; we also hypothesized that when acute exercise occurred in multiple phases, this would have an additive effect on memory.

## Method

### Study Design

All three experiments were approved by the authors’ institutional review board. Participant consent was obtained by all participants prior to any data collection. All three experiments employed within-subject, counterbalanced, experimental designs. Experiment 1 involved six visits to our laboratory, with each visit occurring 48–72 hours apart. Experiments 2 and 3 involved two visits to our laboratory, with each visit occurring 48–72 hours apart. For each of the experiments, the participants subsequent condition did not start until finishing the prior condition (e.g., the subsequent condition did not occur until after they completed the 24-hour memory follow-up assessment of the previous condition). The protocol for each of the experiments is noted below.

#### Experiment 1

See Table 1 (below) for the schematic of the study protocol for Experiment 1.

**Table 1:** Study Protocol for Experiment 1

C	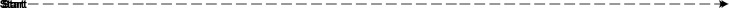							
1	Exercise—20 minutes + 5 minute rest	Memory encoding	55-minute rest^c^			Memory retrieval 1	24-hour rest^a^	Memory retrieval 2^b^
2	Rest—25 minutes^c^	Memory encoding	55-minute rest^c^			Memory retrieval 1	24-hour rest^a^	Memory retrieval 2^b^
3	Rest—25 minutes^c^	Memory encoding	20-minute exercise	35 minute rest^c^		Memory retrieval 1	24-hour rest^a^	Memory retrieval 2^b^
4	Rest—25 minutes^c^	Memory encoding	30 minute rest^c^	20-minute exercise	5-minute rest	Memory retrieval 1	24-hour rest^a^	Memory retrieval 2^b^
5	Exercise—20 minutes + 5 minute rest	Memory encoding	20-minute exercise	35 minute rest^c^		Memory retrieval 1	24-hour rest^a^	Memory retrieval 2^b^
6	Exercise—20 minutes + 5 minute rest	Memory encoding	30 minute rest^c^	20-minute exercise	5-minute rest	Memory retrieval 1	24-hour rest^a^	Memory retrieval 2^b^

*Note.* C = Condition.

^a^This did not occur in the laboratory—the participant left the laboratory for this period and then returned to the lab. No vigorous exercise during this rest period. ^b^This assessment did not occur in the laboratory—participants were called on their phone and asked to verbally complete the memory task. ^c^Participants self-selected a video (DVD of either the Big Bang Theory or The Office) to watch during this resting period. There is experimental evidence that this control/resting period does not alter memory performance (Blough & Loprinzi, 2019).

**Research question**: Does acute exercise enhance memory function via its effects on memory encoding, consolidation or retrieval?

- If the effect is through “encoding,” then Condition 1 > Condition 2
- If the effect is through “consolidation,” then Condition 3 > Condition 2
- If the effect is through “retrieval,” then Condition 4 > Condition 2

**Research question**: Is there an additive effect in enhancing memory if exercise occurs during multiple phases of memory?

- If exercise can enhance both encoding and consolidation additively, more so than encoding alone, then Condition 5 > Condition 1
- If exercise can enhance both encoding and consolidation additively, more so than consolidation alone, then Condition 5 > Condition 3
- If exercise can enhance both encoding and retrieval additively, more so than encoding alone, then Condition 6 > Condition 1
- If exercise can enhance both encoding and retrieval additively, more so than retrieval alone, then Condition 6 > Condition 4

#### Experiment 2

See Table 2 (below) for the schematic of the study protocol for Experiment 2.

**Table 2:** Study Protocol for Experiment 2

C	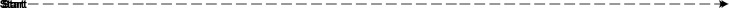						
1	Rest—25 minutes^c^	Memory encoding	4-hour 20-minute rest^a^		Memory retrieval 1	24-hour rest^a^	Memory retrieval 2^b^
2	Rest—25 minutes^c^	Memory encoding	20-minute exercise	4-hour rest^a^	Memory retrieval 1	24-hour rest^a^	Memory retrieval 2^b^

*Note.* C = Condition.

^a^This did not occur in the laboratory—the participant left the laboratory for this period and then returned to the lab. No vigorous exercise during this rest period. ^b^This assessment did not occur in the laboratory—participants were called on their phone and asked to verbally complete the memory task. ^c^Participants self-selected a video (DVD of either the Big Bang Theory or The Office) to watch during this resting period. There is experimental evidence that this control/resting period does not alter memory performance (Blough & Loprinzi, 2019).

**Research question**: When acute exercise occurs during the early consolidation period, does acute exercise enhance memory function via its effects on memory consolidation?

- If the effect is through “consolidation,” then Condition 2 > Condition 1

#### Experiment 3

See Table 3 (below) for the schematic of the study protocol for Experiment 3.

**Table 3:** Study Protocol for Experiment 3

C	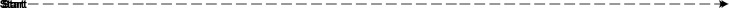							
1	Rest—25 minutes^c^	Memory encoding	4-hour rest^a^			Memory retrieval 1	24-hour rest^a^	Memory retrieval 2^b^
2	Rest—25 minutes^c^	Memory encoding	2-hour rest^a^	20-minute exercise	1-hour 40-minute rest^a^	Memory retrieval 1	24-hour rest^a^	Memory retrieval 2^b^

*Note.* C = Condition.

^a^This did not occur in the laboratory—the participant left the laboratory and then returned to the lab. No vigorous exercise during this rest period. ^b^This assessment did not occur in the laboratory—participants were called on their phone and asked to verbally complete the memory task. ^c^Participants self-selected a video (DVD of either the Big Bang Theory or The Office) to watch during this resting period. There is experimental evidence that this control/resting period does not alter memory performance (Blough & Loprinzi, 2019).

**Research question**: When acute exercise occurs during the late(r) consolidation period, does acute exercise enhance memory function via its effects on memory consolidation?

- If the effect is through “consolidation,” then Condition 2 > Condition 1

### Participants

Participants were excluded (not allowed to participate) if they: (1) were outside the age range of 18–26 y, (2) exercised within 5 hours of their visit, (3) consumed caffeine within 3 hours of their visit, (4) were a daily smoker, (5) self-reported being pregnant, (6) took marijuana in the past 30 days, (7) consumed more than 1 alcoholic drink/day (female) or more than 2 alcoholic drinks/day (male), or (8) had a concussion in the past 30 days.

### Memory Function

For each of the three experiments, participants completed a word-list memory task, involving viewing 15 words on a computer screen, one word at a time, with each word presented, in a set random order, for 1.5 seconds. Participants completed 5 consecutive trials of this (same words per trial), with no recall immediately after encoding. Verbal recall only occurred during Memory Retrieval 1 and Memory Retrieval 2 (see Tables 1–3 for these time-period assessments). After memory encoding, participants were asked to not rehearse the words. This design (no retrieval immediately after encoding) was intentionally implemented, to isolate encoding, consolidation, and retrieval. That is, if participants recalled the words after each of the 5 consecutive trials, then it would be challenging to isolate any potential effects of acute exercise on encoding-related mechanisms (Labban & Etnier, 2018).

A separate word list was used for each visit (condition), with no repeating words for any of the experiments (or conditions). The word lists were composed of 15 words, selected from the MRC Psycholinguistic Database (Wilson, 1988). Each list was matched by concreteness, familiarity and imageability. All words were nouns, had concreteness, imageability, and familiarity ratings between 530 and 700, included 5–10 letters, and 1–2 syllables.

### Exercise Protocol

Participants exercised (vigorous-intensity) for 20-minutes on a treadmill at 75% of heart rate reserve (HRR). This duration and intensity were selected as they are similar to other exercise protocols that have demonstrated a memory enhancement effect (Frith, Sng, & Loprinzi, 2017). After the acute bout of exercise, participants rested (sat) for 5-minutes before starting the memory task. This rest period was selected based on previous work (Frith et al., 2017). Further, this 5-minute rest period was chosen as, when compared to longer recovery periods, levels of key arousal-based memory-related markers (e.g., norepinephrine) are elevated (Skriver et al., 2014; Weiss, Venezia, & Smith, 2019). Thus, we wanted to ensure that the post-exercise memory task occurred prior to these biomarkers returning back to baseline levels.

The HRR equation is:

HRR = [(HR_max_ - HR_rest_) * % intensity] + HR_rest_

To calculate HR_rest_, participants sat quietly for three minutes and HR was recorded from a Polar HR monitor at minute three. To estimate HR_max_, we calculated the participants estimated HR_max_ from 5 commonly used equations to estimate HR_max_. We took the average of these 5 estimates and used this average in the above HRR equation. The 5 HR_max_ equations that were used include:

Fox: 220-age

Astrand: 216.6 – (0.84*age)

Tanaka: 208 – (0.7*age)

Gellish: 207 – (0.7*age)

Gulati: 206 – (0.88*age)

Throughout the treadmill exercise, HR was continuously monitored using a Polar HR monitor, and HRs were recorded at baseline, midpoint, endpoint, and 5-minutes post-exercise.

### Statistical Analyses

JASP statistical software was utilized to analyze the data. For Experiment 1, a 2 (Time: Memory Retrieval 1 and 2) by 6 (Condition) ANOVA (analysis of variance) was employed to evaluate memory performances across the six conditions. For Experiments 2 and 3, a 2 (Time: Memory Retrieval 1 and 2) by 2 (Condition) ANOVA was employed to evaluate memory performances across the two conditions. Main effects (time and condition) and interactions are reported, with effect size estimates reported as eta-squared values (η^2^). Statistical significance was set at an alpha of .05. We intentionally did not correct for multiple comparisons, as the number of type I errors cannot decrease without increasing the risk of making a type II error. Further, the theoretical assumption behind correction for multiple testing is that all null hypotheses are true simultaneously, which was not of interest in our study (Perneger, 1998; Rothman, 1990).

## Results

### Participant Characteristics

Demographic and behavioral results of the participants in the three experiments are shown below (Table 4). The sample sizes for Experiments 1–3, respectively, were 47, 42, and 31. This was based on prior work demonstrating that a sample size of at least 30 would be needed to elicit a power (1—β error probability) of .80 for an expected η^2^ of .08 (Loprinzi et al., 2019).

**Table 4:** Participant Characteristics Across the Three Experiments

Variable	Experiment 1	Experiment 2	Experiment 3
Age, mean years	21.1 (1.7)	20.6 (1.1)	20.5 (1.0)
Gender, % female	57.4	53.7	87.1
Race-Ethnicity, % White	76.6	90.2	70.9
BMI, mean kg/m^2^	25.1 (4.5)	24.0 (4.3)	24.2 (4.3)
MVPA, mean minutes/week	228.2 (260.9)	176.7 (151.4)	157.7 (137.4)

*Note.* BMI = body mass index; MVPA = moderate-to-vigorous physical activity (self-reported from the Physical Activity Vital Signs questionnaire). Variance estimates are standard deviations.

### Physiological Responses to Exercise/Rest Conditions

Table 5 displays the heart rate responses to the exercise and control conditions. All exercise bouts resulted in a similar physiological response.

**Table 5:** Physiological (Heart Rate) Responses to the Exercise and Rest Conditions Across the Three Experiments

Experiment	Rest		Midpoint		Endpoint		Post	
	*M*	*SD*	*M*	*SD*	*M*	*SD*	*M*	*SD*
Experiment 1								
Condition 1 (Exercise)	80.4	11.4	155.3	16.8	164.2	7.2	97.8	10.1
Condition 2 (Rest)	75.4	10.3	71.6	10.0	71.8	11.6	72.8	8.2
Condition 3 (Exercise)	74.8	10.2	153.4	17.0	164.0	7.0	93.4	11.7
Condition 4 (Exercise)	74.6	11.8	154.0	18.4	164.3	7.4	97.4	11.9
Condition 5—Bout 1 (Exercise)	79.5	11.5	153.5	17.3	164.3	10.2	99.1	12.7
Condition 5—Bout 2 (Exercise)	89.2	11.3	157.4	14.8	164.1	8.5	96.8	12.9
Condition 6—Bout 1 (Exercise)	79.1	11.4	153.3	17.7	163.8	9.6	97.6	9.6
Condition 6—Bout 2 (Exercise)	80.1	9.9	154.7	16.7	164.6	7.3	99.2	10.4
Experiment 2^a^								
Condition 2	78.6	10.3	160.1	8.1	172.2	5.0	-	-
Experiment 3^a^								
Condition 2	78.2	10.2	161.3	12.4	166.5	8.3	92.3	10.3

*Note.* “-“ means not assessed.

^a^Heart rate only assessed in the exercise condition (i.e., Condition 2) for Experiments 2 and 3 (i.e., not assessed during the resting condition).

### Memory Outcomes

#### Experiment 1

**Table:** 

C	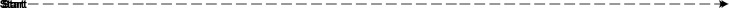							
1	Exercise—20 minutes + 5 minute rest	Memory encoding	55-minute rest^c^			Memory retrieval 1	24-hour rest^a^	Memory retrieval 2^b^
2	Rest—25 minutes^c^	Memory encoding	55-minute rest^c^			Memory retrieval 1	24-hour rest^a^	Memory retrieval 2^b^
3	Rest—25 minutes^c^	Memory encoding	20-minute exercise	35 minute rest^c^		Memory retrieval 1	24-hour rest^a^	Memory retrieval 2^b^
4	Rest—25 minutes^c^	Memory encoding	30 minute rest^c^	20-minute exercise	5-minute rest	Memory retrieval 1	24-hour rest^a^	Memory retrieval 2^b^
5	Exercise—20 minutes + 5 minute rest	Memory encoding	20-minute exercise	35 minute rest^c^		Memory retrieval 1	24-hour rest^a^	Memory retrieval 2^b^
6	Exercise—20 minutes + 5 minute rest	Memory encoding	30 minute rest^c^	20-minute exercise	5-minute rest	Memory retrieval 1	24-hour rest^a^	Memory retrieval 2^b^

*Note.* C = Condition.

^a^This did not occur in the laboratory—the participant left the laboratory for this period and then returned to the lab. No vigorous exercise during this rest period. ^b^This assessment did not occur in the laboratory—participants were called on their phone and asked to verbally complete the memory task. ^c^Participants self-selected a video (DVD of either the Big Bang Theory or The Office) to watch during this resting period. There is experimental evidence that this control/resting period does not alter memory performance (Blough & Loprinzi, 2019).

**Figure 1:**
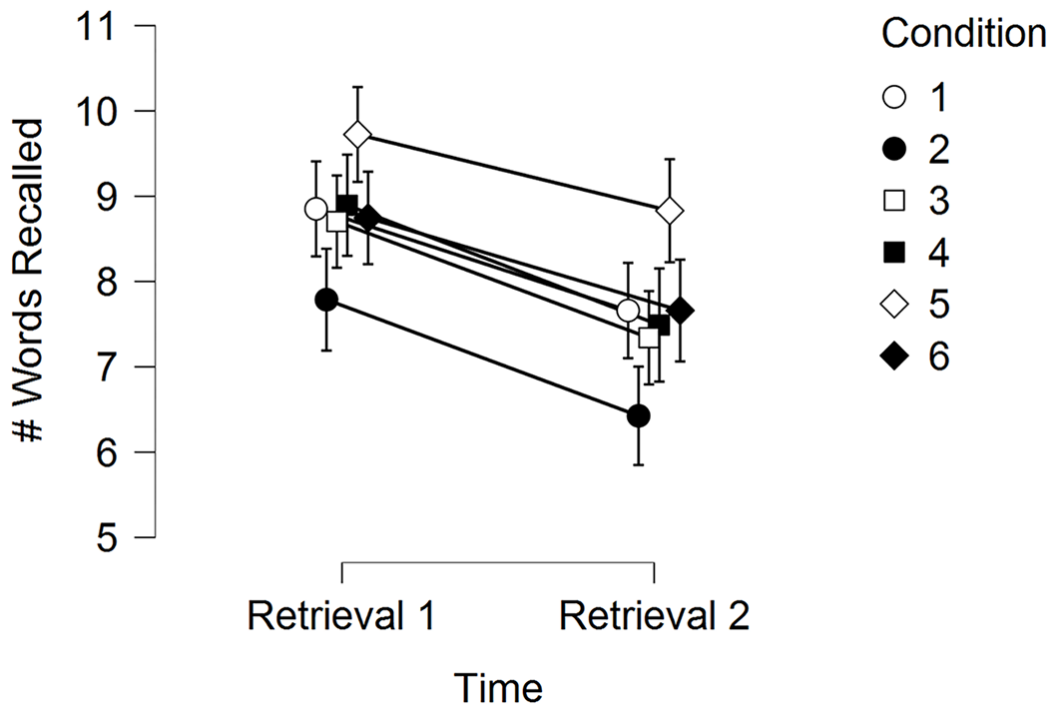
Experiment 1 Memory Performance (Mean Words Recalled) Across Time for the Six Experimental Conditions *Note*. Error bars are standard errors.

Table 6 and Figure 1 display the memory results for Experiment 1.

**Table 6:** Memory Results (Words Recalled) for Experiment 1

Condition	Memory retrieval 1		Memory retrieval 2	
	*M*	*SD*	*M*	*SD*
1	8.85	3.81	7.66	3.82
2	7.78	4.09	6.42	3.95
3	8.70	3.70	7.34	3.74
4	8.89	4.06	7.48	4.54
5	9.72	3.80	8.83	4.14
6	8.74	3.71	7.66	4.09

In a 2 (Time) × 6 (Condition) RM-ANOVA, there was a significant main effect for Time, *F*(1, 46) = 149.18, *p* < .001, η^2^ = .02, main effect for Condition, *F*(5, 230) = 5.32, *p* < .001, η^2^ = .02, but no Time by Condition interaction, *F*(5, 230) = 0.78, *p* = .56, η^2^ = .001. Since there was no time by condition interaction, memory scores were collapsed across the two time points for each condition, with post-hoc testing comparing these time collapsed condition effects (see Table 7).

**Table 7:** Post-Hoc Results Comparing the Memory Results (Collaposed Across the Two Memory Time Periods) Across the Six Experimental Conditions

Condition comparison	*M*_diff_	*SE*	*t*	*p*
C1				
C2	1.15	0.38	3.01	.004
C3	0.23	0.45	0.51	.60
C4	0.06	0.37	0.17	.86
C5	−1.02	0.33	3.02	.004
C6	0.05	0.41	0.12	.89
C2				
C3	−0.91	0.48	1.90	.06
C4	−1.08	0.46	2.32	.02
C5	−2.17	0.35	6.15	< .001
C6	−1.09	0.38	2.88	.006
C3				
C4	−0.17	0.46	0.36	.71
C5	−1.25	0.48	2.58	.01
C6	−0.18	0.43	0.41	.67
C4				
C5	−1.08	0.39	2.78	.007
C6	−0.01	0.48	0.02	.98
C5				
C6	1.07	0.40	2.65	.01

*Note.* C = Condition.

Table 8 indicates the results for each specific research question.

**Table 8:** Summary of Results for Experiment 1

Research question	Yes	No	Maybe	Notes
**Did acute exercise enhance memory?**	X			Condition 2 (resting) had the lowest memory performance
If the effect is through “encoding,” then Condition 1 > Condition 2	X			Condition 1 was > Condition 2 (*p* = .004)
If the effect is through “consolidation,” then Condition 3 > Condition 2			X	Condition 3 was not quite statistically significantly different than Condition 2 (*p* = .06)
If the effect is through “retrieval,” then Condition 4 > Condition 2	X			Condition 4 was > Condition 2 (*p* = .02)
**Is there an additive effect in enhancing memory if exercise occurs during multiple phases of memory?**	X			When acute exercise occurred in multiple phases of memory, memory performance was enhanced.
If exercise can enhance both encoding and consolidation additively, more so than encoding alone, then Condition 5 > Condition 1	X			Condition 5 was > Condition 1 (*p* = .004)
If exercise can enhance both encoding and consolidation additively, more so than consolidation alone, then Condition 5 > Condition 3	X			Condition 5 was > Condition 3 (*p* = .01)
If exercise can enhance both encoding and retrieval additively, more so than encoding alone, then Condition 6 > Condition 1		X		Condition 6 was not > Condition 1 (*p* = .89)
If exercise can enhance both encoding and retrieval additively, more so than retrieval alone, then Condition 6 > Condition 4		X		Condition 6 was not > Condition 4 (*p* = .98)

As shown in Table 8 above, acute exercise was effective in enhancing memory function. There was evidence that this effect may have occurred through mechanisms related to encoding and retrieval. There was also some suggestive evidence (*p* = .06) that exercise may enhance memory via consolidation-based mechanisms. Experiments 2 and 3 further evaluates this potential effect and how the timing of exercise during the consolidation period may influence whether exercise enhances memory via consolidation-based mechanisms. First, Experiment 2 extends Experiment 1 by extending the consolidation period. In Experiment 1, for Condition 3, there was only a 35-minute consolidation period after the acute bout of exercise. This may have been too short to observe any beneficial effects of acute exercise during the early consolidation period. As such, in Experment 2, we extended the post-exercise consolidation period from 35-minutes to 4-hours.

#### Experiment 2

**Table:** 

C	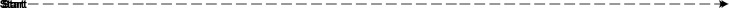						
1	Rest—25 minutes^c^	Memory encoding	4-hour 20-minute rest^a^		Memory retrieval 1	24-hour rest^a^	Memory retrieval 2^b^
2	Rest—25 minutes^c^	Memory encoding	20-minute exercise	4-hour rest^a^	Memory retrieval 1	24-hour rest^a^	Memory retrieval 2^b^

*Note.* C = Condition.

^a^This did not occur in the laboratory—the participant left the laboratory for this period and then returned to the lab. No vigorous exercise during this rest period. ^b^This assessment did not occur in the laboratory—participants were called on their phone and asked to verbally complete the memory task. ^c^Participants self-selected a video (DVD of either the Big Bang Theory or The Office) to watch during this resting period. There is experimental evidence that this control/resting period does not alter memory performance (Blough & Loprinzi, 2019).

**Figure 2:**
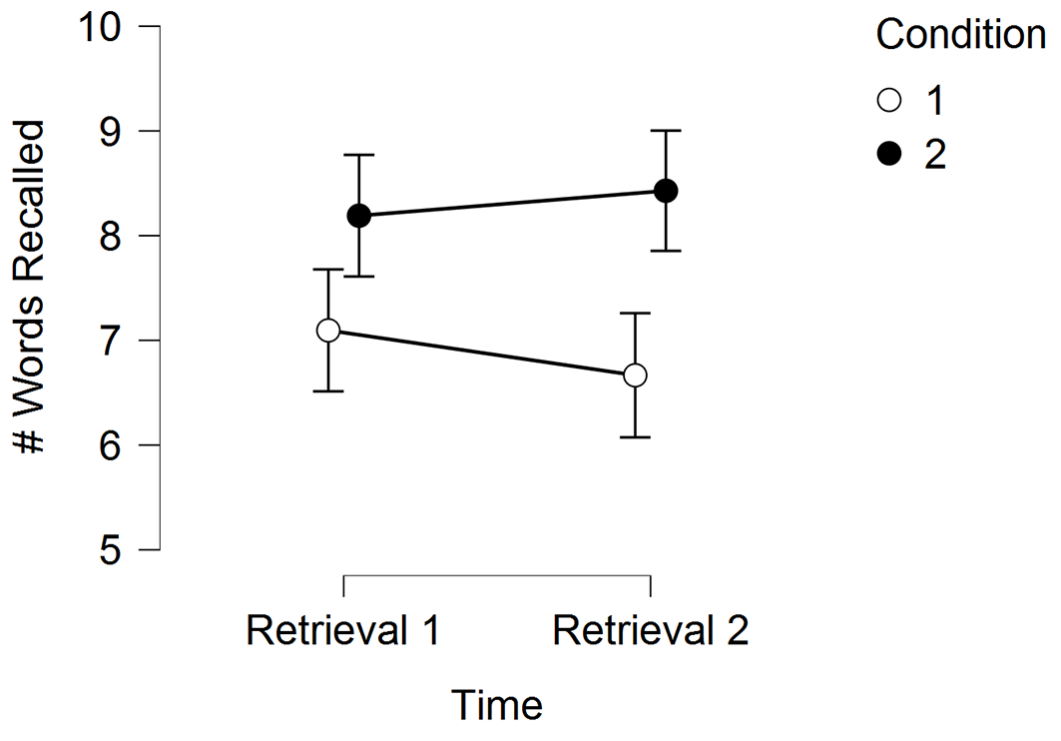
Experiment 2 memory performance (mean words recalled) across time for the two experimental conditions *Note*. Error bars are standard errors.

Table 9 and Figure 2 display the memory results for Experiment 2.

**Table 9:** Memory Results (Words Recalled) for Experiment 2

Condition	Memory retrieval 1		Memory retrieval 2	
	*M*	*SD*	*M*	*SD*
1	7.09	3.77	6.66	3.84
2	8.20	3.72	8.41	3.67

In a 2 (Time) × 2 (Condition) RM-ANOVA, there was not a significant main effect for Time, *F*(1, 41) = 0.42, *p* = .52, η^2^ = .001, but there was a significant main effect for Condition, *F*(1, 41) = 4.58, *p* = .04, η^2^ = .09, and a significant Time by Condition interaction, *F*(1, 41) = 5.34, *p* = .03, η^2^ = .005. Post-hoc tests are shown in Table 10.

**Table 10:** Post-Hoc Results Comparing the Memory Results Across the Experimental Conditions (Results Evaluated Across Condition and Time Period)

Condition comparison	*M*_diff_	*SE*	*t*	*p*
C1, R1				
C1, R2	0.43	0.18	2.37	.02
C2, R1	−1.09	0.69	1.57	.12
C2, R2	−1.33	0.67	1.96	.06
C1, R2				
C2, R1	−1.52	0.68	2.22	.03
C2, R2	−1.76	0.67	2.62	.01
C2, R1				
C2, R2	−0.20	0.22	0.93	.35

*Note*. C = Condition; R = Retrieval.

Table 11 indicates the results for the specific research question for Experiment 2.

**Table 11:** Summary of Results for Experiment 2

Research question	Yes	No	Maybe	Notes
**When acute exercise occurs during the early consolidation period, does acute exercise enhance memory function via its effects on memory consolidation?**	X			
If the effect is through “consolidation,” then Condition 2 > Condition 1	X			Condition 2 was > Condition 1 (*p* = .04) (main effect for condition)^a^

^a^However, this effect was only significant at Memory retrieval 2 (*p* = .01) and not Memory retrieval 1 (*p* = .12). Thus, for acute exercise during the immediate consolidation period to have any effect on enhancing memory, a long follow-up period (over 4-hours) may be needed.

As shown in Table 11 above, acute exercise was effective in enhancing memory function. This is in contrast to the results (Condition 3 vs. Condition 2) in Experiment 1. For Condition 3 in Experiment 1, there was only a 35-minute consolidation period after the acute bout of exercise. This may have been too short to observe any benefiical effects of acute exercise during the early consolidation period. As such, in Experment 2, we extended the post-exercise consolidation period from 35-minutes to 4-hours. Our results from Experiment 2 suggest that for acute exercise during the immediate consolidation period to have any effect on enhancing memory, a long follow-up period (over 4-hours) may be needed.

Experiment 3 builds off of Experiment 2 by evaluating whether altering the timing of the acute bout of exercise during the consolidation period has an effect on long-term memory function. That is, instead of exercising immediately after encoding, in Experiment 3, the acute bout of exercise occurs 2-hours after memory encoding.

#### Experiment 3

**Table:** 

C	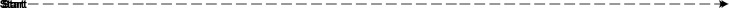							
1	Rest—25 minutes^c^	Memory encoding	4-hour rest^a^			Memory retrieval 1	24-hour rest^a^	Memory retrieval 2^b^
2	Rest—25 minutes^c^	Memory encoding	2-hour rest^a^	20-minute exercise	1-hour 40-minute rest^a^	Memory retrieval 1	24-hour rest^a^	Memory retrieval 2^b^

*Note.* C = Condition.

^a^This did not occur in the laboratory—the participant left the laboratory and then returned to the lab. No vigorous exercise during this rest period. ^b^This assessment did not occur in the laboratory—participants were called on their phone and asked to verbally complete the memory task. ^c^Participants self-selected a video (DVD of either the Big Bang Theory or The Office) to watch during this resting period. There is experimental evidence that this control/resting period does not alter memory performance (Blough & Loprinzi, 2019).

**Figure 3:**
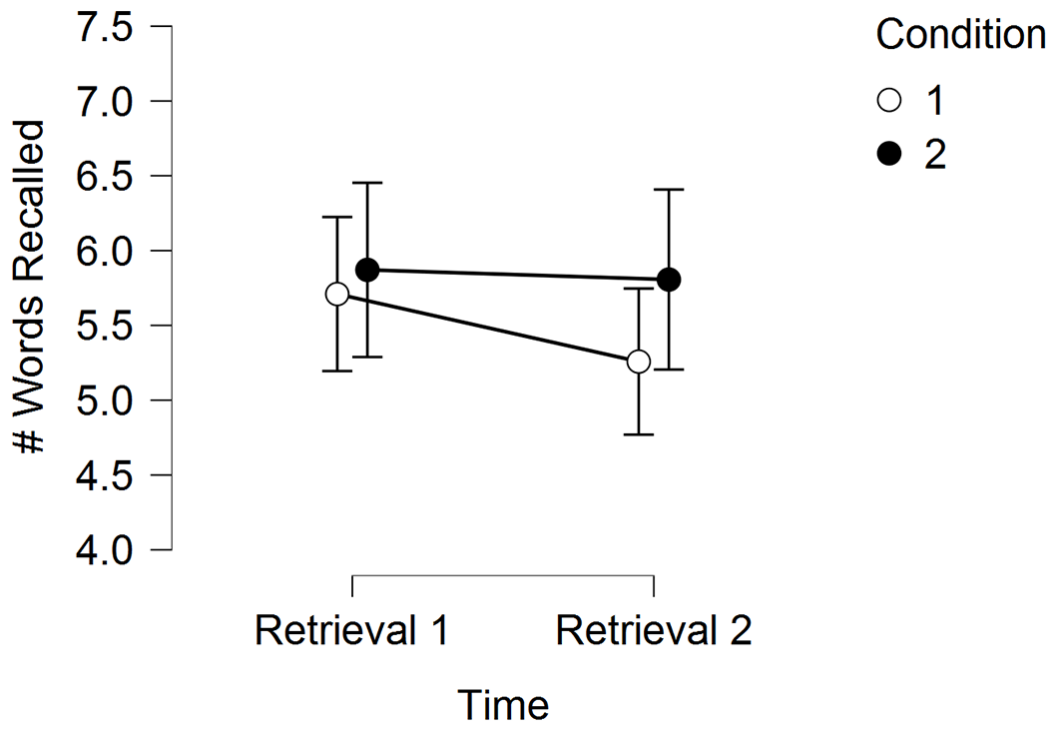
Experiment 3 memory performance (mean words recalled) across time for the two experimental conditions *Note*. Error bars are standard errors.

Table 12 and Figure 3 display the memory results for Experiment 3.

**Table 12:** Memory Results (Words Recalled) for Experiment 3

Condition	Memory retrieval 1		Memory retrieval 2	
	*M*	*SD*	*M*	*SD*
1	5.71	2.86	5.25	2.72
2	5.87	3.24	5.80	3.35

In a 2 (Time) × 2 (Condition) RM-ANOVA, there was not a significant main effect for Condition, *F*(1, 30) = 0.52, *p* = .47, η^2^ = .003, but there was a significant main effect for Time, *F*(1, 30) = 5.18, *p* = .03, η^2^ = .002, and a marginally significant Time by Condition interaction, *F*(1, 30) = 2.94, *p* = .10, η^2^ = .001. Post-hoc tests are shown in Table 13.

**Table 13:** Post-Hoc Results Comparing the Memory Results Across the Experimental Conditions (Results Evaluated Across Condition and Time Period)

Condition comparison	*M*_diff_	*SE*	*t*	*p*
C1, R1				
C1, R2	0.45	0.15	2.95	.006
C2, R1	−0.16	0.52	0.30	.75
C2, R2	−0.09	0.50	0.19	.84
C1, R2				
C2, R1	−0.61	0.50	1.20	.23
C2, R2	−0.54	0.48	1.12	.26
C2, R1				
C2, R2	0.06	0.16	0.38	.70

*Note*. C = Condition; R = Retrieval.

Table 14 indicates the results for the specific research question for Experiment 3.

**Table 14:** Summary of Results for Experiment 3

Research question	Yes	No	Maybe	Notes
**When acute exercise occurs during the late(r) consolidation period, does acute exercise enhance memory function via its effects on memory consolidation?**		X		
If the effect is through “consolidation,” then Condition 2 > Condition 1		X		Condition 2 was not > Condition 1 (*p* = .47) (main effect for condition)^a^

^a^Thus, when the acute bout of exercise occurs 2 hours after memory encoding, long-term memory is not enhanced from exercise. This is in contrast to the results from Experiment 2, which showed that when acute exercise occurred immediately after encoding, long-term memory was enhanced. Thus, the timing of acute exercise during the consolidation period may play an important role.

As shown above in Table 14, when the acute bout of exercise occurred 2-hours after memory encoding, long-term memory function was not enhanced.

## Discussion

### Study Objectives

The present set of experiments were conducted to answer a series of important questions. First, we set out to evaluate whether acute exercise can enhance short- and long-term memory. Previous work has already attempted to answer this question, but the findings in the literature are considerably mixed, with about half of the previous studies demonstrating an acute exercise-induced enhancement effect of memory (Loprinzi et al., 2019; Roig et al., 2013). Given these mixed findings, re-addressing this potential relationship is a worthwhile endeavor. Second, a novel aspect of our experiments was the systematic investigation of whether acute exercise enhanced memory through encoding, consolidation or retrieval-based mechanisms. Third, we evaluated whether exercise, occurring across multiple phases of memory (before encoding and during consolidation), can additively influence memory function.

### Main Findings

The main findings of our experiments are as follows. First, we demonstrated the ability for high-intensity acute exercise to enhance memory function. Second, the exercise-induced improvement in memory may have occurred through mechanisms related to encoding and retrieval. As shown in our Experiment 1 results, when exercise occured both before encoding and during consolidation, memory was enhanced to a greater extent than when exercise occured in just one of these temporal periods. In Experiment 1, we also demonstrated some suggestive evidence (*p* = .06) that exercise may enhance memory via consolidation-based mechanisms. Experiment 2 demonstrated that acute exercise during early consolidation likely has a greater effect when there has been at least 4 hours for the memory to consolidate (i.e., there was no effect at 4-hours, but there was an effect at 24-hours). Experiment 3 demonstrated that any effect of acute exercise during consolidation is likely to occur if the acute bout of exercise occurs during the early consolidation period as opposed to the acute bout of exercise occurring during the late consolidation period. Notably, however, Experiments 2 and 3 demonstrate that when acute exercise occurs during the consolidation period, it may attenuate forgetting (see Figures 2 and 3 for an attenuated decay in memory for the exercise condition). We further discuss each of these main findings in the narrative that follows.

### Acute Exercise on Memory

As indicated in the first paragraph of this Discussion section, only about half of the prior studies on this topic have demonstrated that acute exercise can enhance memory function. This inconsistent relationship is not surprising, as the acute exercise-memory interaction is a complex relationship, likely influenced by a multitude of factors. For example, it is plausible that the physiological response to the acute exercise may moderate this relationship. Such a physiological response may vary as a function of the duration and/or intensity of the exercise stimulus, the age of the participant, and/or the fitness level of the individual (Pontifex et al., 2019). Other potential factors may include, for example, the timing of the exercise bout to the memory stimulus and the memory type evaluated (e.g., emotional episodic memory, non-emotional episodic memory, semantic memory, implicit memory, procedural memory). Several researchers (Loprinzi et al., 2019; Roig et al., 2013) have attempted to answer how some of these factors may influence the acute exercise-memory relationship. However, at this point, it is still unclear as to the extent to which these factors may truly moderate the effects of acute exercise on memory performance. As such, a lot of additional research on this topic is needed.

Our experimental results herein demonstrate that high-intensity acute exercise was effective in enhancing memory function among this relatively active, young-adult population. This was a robust effect, occurring across multiple experimental conditions for Experiments 1 and 2. This aligns with a past review suggesting greater beneficial effects of acute exercise on memory in young (vs. old) populations (Loprinzi et al., 2019). Previous research has speculated, in detail, on the mechanisms of this potential relationship (El-Sayes et al., 2019; Loprinzi et al., 2017, 2018; Pontifex et al., 2019). In brief, acute exercise may influence memory as a result of exercise-induced alterations in various neurotransmitters and growth factors that influence long-term potentiation (Loprinzi, 2019). Of course, and as discussed in the following section, the mechanisms of this potential relationship are likely to be very complex and comprehensive, and may vary based on the memory phase (encoding, consolidation, and retrieval).

### Does Acute Exercise Enhance Memory via Encoding, Consolidation, or Retrieval-Based Mechanisms?

The answer to this research question is addressed from all three of our experiments. As shown in Table 8, Condition 1 was > Condition 2, suggesting that exercise may influence memory via encoding-related mechanisms. Reasons for this effect has been speculated elsewhere (Loprinzi et al., 2018). For example, acute exercise may enhance attention, and as such, may facilitate the allocation of resources to encoding the memory stimuli, and ultimately, the acquisition of the memory trace (engram). Relatedly, and from a neurophysiological perspective, acute exercise may increase neuronal cAMP response element-binding protein (CREB) phosphorylation, and as a result, prime specific neural networks to be allocated to the memory engram (i.e., the population of neurons that represents the memory). Future experimental work is needed to specifically evaluate these speculated mechanisms. Such work, when feasible, should be carried out in human populations.

As shown in Table 8, Condition 4 was > Condition 2, suggesting that exercise may influence memory via retrieval-based mechanisms. The specific retrieval-based mechanisms of this potential effect are more difficult to explain. Elsewhere (Loprinzi et al., 2017), researchers have speculated that this may occur via facilitating the item-invariant component of memory retrieval. That is, acute exercise may facilitate mental resources that may be needed for searching, monitoring and controlling the processes involved in locating and retrieving a memory. However, it is unclear, to us, as to how exercise would, indeed, accomplish this. Exercise may, potentially, accomplish this by facilitating executive function. If our observed effect is replicated in future research, then critical evaluation will be needed to understand how acute exercise may enhance memory via retrieval-based mechanisms. We leave this speculation and critical reflection to future papers isolated on this specific effect.

As shown in Table 8, our results from Experiment 1 provide suggestive evidence (*p* = .06) that acute exercise may enhance memory via consolidation-based mechanisms. In Experiment 1, Condition 3 was almost (*p* = .06) > than Condition 2, suggesting that acute exercise immediately after encoding may enhance memory when memory is assessed 35-minutes post-exercise. Experiment 2 also demonstrated that memory may be enhanced when acute exercise occurs immediately after encoding. Importantly, however, this effect was only observed for the 24-hour follow-up assessment. Thus, when acute exercise occurs immediately after encoding, it may influence memory function, but likely will not influence memory if the subsequent memory assessment occurs somewhere between 35-minutes (Experiment 1) and 4-hours (Experiment 2). It seems that the beneficial effects of acute exercise immediately after encoding may require a lengthy consolidation period. Of course, at this point, the reason for this potential effect is not known. Our results from both Experiment 2 and Experiment 3 suggest that acute exercise during the early consolidation period may, potentially, attenuate memory decay. That is, the slope between Retrieval 1 and Retrieval 2 had a visually steeper decline in the control condition when compared to the exercise condition. This finding, however, is in contrast to a recent meta-analysis showing that when exercise occurs prior to memory encoding, exercise, compared to control, does not attenuate forgetting (Moore, Ryu, & Loprinzi, 2020). Importantly, however, unlike Experiment 2, this prior meta-analysis evaluated studies that implemented the exercise bout before encoding, as opposed to during consolidation. Perhaps the lengthier consolidation period that is needed to observe a beneficial effect of acute exercise during the early consolidation is due to this potential exercise-induced attenuation of memory decay. This also could be related to our memory task, which involved a 15-word stimulus. Perhaps a more difficult memory stimulus would have resulted in greater variability at Retrieval 1. Thus, at this point, we cannot confidently conclude that a 4+ hour consolidation period is needed to observe exercise-induced memory enhancement effects when the bout of exercise occurs immediately after encoding. Perhaps, based on the employed memory task, beneficial effects may occur within a shorter consolidation window. Of course, in order to gain a better understanding of this time frame in which exercise may benefit memory, it would first be important to identify how long it would take for the memory to be consolidated. This, however, is a challenging task, as, at this point, it is unclear, within the human population, as to how long it takes for memories to consolidate. This may vary as a function of several factors, such as the extent of encoding or the degree of interfering stimuli during the consolidation window. We also need a better understanding of the underlying exercise-induced mechanisms that would help facilitate memory consolidation. As discussed elsewhere (Loprinzi et al., 2018), various neurotransmitters, such as norepinephrine, acetylcholine, serotonin, and dopamine may be implicated in exercise-induced memory consolidation. Importantly, however, any exercise-induced increases in these neurotransmitters would return to baseline long before the entire consolidation window. Thus, it is unclear, from a mechanistic perspective, as to how a very short (e.g., 20-minutes) bout of exercise during the early consolidation period would have a sustained effect on memory consolidation. This line of thinking may benefit from previous thoughts on how long-term memories may outlast the life of proteins that play a critical role in the consolidation and maintenance of the memory. That is, after the acquisition of the engram, the engram, and its related proteins and enzymes (e.g., kinases), may, theoretically, enter a stable state (via positive feedback loops) by regeneration occurring repeatedly. Perhaps acute exercise, in some way, facilitates this effect. Critically, however, perhaps there is a necessary window for this to occur. It is likely that the optimal window is shortly after the acquisition of the memory trace (after encoding), prior to any substantial decay in the engram. Our results align with these speculations, as we did not observe any beneficial effects on memory when the acute bout of exercise occurred 2-hours after memory encoding.

### If Acute Exercise Occurs During Multiple Memory Phases, Does it Additively Influence Memory?

Another noteworthy and interesting observation of our results was that acute exercise additively influenced memory when multiple bouts of exercise occurred across multiple phases of memory. To our knowledge, this concept was first introduced by Roig and colleagues (Roig et al., 2016) and subsequently tested in a recent publication (Loprinzi, Chism, & Marable, 2019). Loprinzi et al. integrated an acute bout of exercise both before encoding and during early consolidation when compared to a condition of exercise only prior to encoding. In this prior experiment (Loprinzi, Chism, & Marable, 2019), however, there were no differential effects on memory between these two aforementioned conditions. However, in this prior experiment, a moderate-intensity bout of acute exercise was used, which may have been too low of a physiological stimulus to enhance memory (Loprinzi, 2018). In the present experiment, we implemented a high-intensity bout of exercise and demonstrated that exercise may additively influence memory if it occurs during multiple phases of memory. This also aligns with a recent experiment showing that stationary cycling (55-65% of heart rate reserve) for 10 minutes both before and after memory encoding was more effective, relative to control, in enhancing long-term memory when compared to cycling for 20 minutes before or after memory encoding (Slutsky-Ganesh et al., 2020). A potential mechanistic explanation for this observation is that, perhaps, exercise activates the unique mechanisms for the respective memory phases (discussed in the above sections as well as in prior reviews; Loprinzi et al., 2017, 2018).

A notable observation of our results (Experiment 1; see Table 8) was that exercise presumably enhanced both encoding and consolidation additively, more so than encoding alone (Condition 5 > Condition 1) or consolidation alone (Condition 5 > Condition 3). However, exercise did not presumably enhance encoding and retrieval additively, more so than encoding alone (Condition 6 was not > Condition 1) or retrieval alone (Condition 6 was not > Condition 4). Collectively, these results suggest that, if an additive effect is a true causal phenomenon, then perhaps the optimal time to exercise would be shortly before memory encoding and during the early consolidation period, as opposed to exercising before memory retrieval. This, of course, is regarding multiple bouts of exercise, as opposed to a single bout of exercise, which we demonstrated in Experiment 1 that exercising shortly before memory retrieval may enhance memory (Condition 4 > Condition 2).

### Limitations

It is important to interpret the present set of findings within the context of the limitations of these experiments. We employed a homogenous sample of young adults. Thus, our findings cannot be extrapolated to other populations. Even within this young adult population, it may be inappropriate to generalize our findings to individuals with other behavioral characteristics, such as a less physically active group. Second, as demonstrated by the eta-squared estimates, the observed effect sizes were relatively small, and thus, the meaningfulness of our observed differences should be interpreted accordingly. Although word-list tasks are commonly employed laboratory measures of memory function, perhaps larger effect sizes would be observable with a more comprehensive, integrated assessment of episodic memory. However, other work has previously integrated such comprehensive memory tasks (e.g., that integrate aspects of “what,” “where” and “when” components of episodic memory; Blough & Loprinzi, 2019), and, from these studies, there is considerable participant variability in such tasks, ultimately requiring very large samples to detect relatively small effects. We do feel that future work should carefully reflect on the trade-off between task simplicity and real-life utility. Another limitation is that we did not measure heart rate during the resting conditions for Experiments 2 and 3. Although we recognize this as a limitation, participants were sitting during the entire resting period, and thus, their physiological arousal was substantively lower than the exercise conditions. Further, during the exercise bouts, heart rate was only recorded at the midpoint and endpoint. To provide a greater overall estimate of the physiological response to exercise, a better approach would be to average the heart rates across the entire bout of exercise. Three additional issues are worth considering when interpreting our data. For logistical reasons, our Retrieval 2 assessments took place over the phone, as opposed to in the laboratory. However, we asked participants to be in a quiet room with no distractions, to ensure the participants were able to effectively focus on retrieving as many words as possible. As fully discussed in our Statistical Analysis section, we intentionally chose not to correct for multiple comparisons. All our results report the exact *p*-value, and as such, the cautious reader can interpret our findings in light of our observed *p*-values. Lastly, our imposed acute exercise intensity (75% of HRR) was from an estimate. That is, due to logistical reasons (e.g., those in Experiment 1 already completed 6 laboratory visits), we did not have the participants complete an initial maximal treadmill test to determine their maximal heart rate. However, we did utilize 5 separate equations to best estimate their maximal heart rate.

### Strengths

Notable strengths of this paper are multifold. First, we employed a within-subject, counterbalanced design for all three experiments. This helps overcome the biggest source of memory variance in between-subject designs, which is individual differences in memory function. Secondly, we conducted three integrated experiments to answer several novel research questions. Third, we included long-term (24-hour) follow-up memory assessments, which are infrequently evaluated in exercise-memory experiments.

### Recommendations for Future Research

The primary recommendations we have for future research are two-fold. First, additional replicative work is needed. Second, if our findings ultimately demonstrate to be causal, repeatable effects, then future work should carefully reflect on potential causal mechanisms and appropriately design experiments to evaluate the mediators (e.g., long-term potentiation) of the exercise-memory relationship.

### Conclusion

In conclusion, we demonstrate several notable results from our experiments. First, we demonstrated the ability for high-intensity acute exercise to enhance memory function, and possibly, attenuate forgetting. Second, the exercise-induced improvement in memory may have occurred through mechanisms related to encoding, consolidation and retrieval. Third, incorporating acute exercise into multiple phases of memory may additively enhance memory function.
